# Stochastic Fluctuations and Distributed Control of Gene Expression Impact Cellular Memory

**DOI:** 10.1371/journal.pone.0115574

**Published:** 2014-12-22

**Authors:** Guillaume Corre, Daniel Stockholm, Ophélie Arnaud, Gaël Kaneko, José Viñuelas, Yoshiaki Yamagata, Thi My Anh Neildez-Nguyen, Jean-Jacques Kupiec, Guillaume Beslon, Olivier Gandrillon, András Paldi

**Affiliations:** 1 Inserm, UMR 951, Université d'Evry Val d'Essonne, Genethon, Evry, F91002 France; 2 Ecole Pratique des Hautes Etudes, Paris, France; 3 Université de Lyon, Université Lyon 1, Centre de Génétique et de Physiologie Moléculaire et Cellulaire (CGPhiMC), CNRS UMR5534, F-69622 Lyon, France; 4 Université de Lyon, INSA-Lyon, INRIA, Laboratoire d'InfoRmatique en Image et Systèmes d'information (LIRIS), CNRS UMR5205, F-69621 Lyon, France; 5 Centre Cavailles, Ecole Normale Supérieure, 29 rue d'Ulm, 75005 Paris, France; 6 Department of Obstetrics and Gynaecology, Yamaguchi University Graduate School of Medicine, Ube, Japan; University of Southampton, United Kingdom

## Abstract

Despite the stochastic noise that characterizes all cellular processes the cells are able to maintain and transmit to their daughter cells the stable level of gene expression. In order to better understand this phenomenon, we investigated the temporal dynamics of gene expression variation using a double reporter gene model. We compared cell clones with transgenes coding for highly stable mRNA and fluorescent proteins with clones expressing destabilized mRNA-s and proteins. Both types of clones displayed strong heterogeneity of reporter gene expression levels. However, cells expressing stable gene products produced daughter cells with similar level of reporter proteins, while in cell clones with short mRNA and protein half-lives the epigenetic memory of the gene expression level was completely suppressed. Computer simulations also confirmed the role of mRNA and protein stability in the conservation of constant gene expression levels over several cell generations. These data indicate that the conservation of a stable phenotype in a cellular lineage may largely depend on the slow turnover of mRNA-s and proteins.

## Introduction

Specific gene regulation mechanisms are believed to ensure a constant expression level and guarantee long-term phenotypic stability of the cells and cell lineages. However, gene expression, as biochemical reactions in general, is a probabilistic process [1] essentially because of the low copy number of participating molecules. As a result, mRNA and protein levels in vary widely even between cells of a clonal populations exposed to a homogenous environment. This general phenomenon that concerns every gene in every cell type of multicellular organisms poses a challenge to our understanding of the phenotypic stability of the cell [2]. It has been shown that the energetic costs of the suppression of this noise by specific regulatory mechanisms is very high [3]. This makes impossible the suppression of the fluctuations in individual cells beyond a certain limit. The difficulty is similar if we want to explain how a phenotype can be stably transmitted over cell divisions in a cell lineage. Again, this role is typically attributed to memory mechanisms of gene transcription regulation [4]. However, these mechanisms are also blurred by noise.

**Figure 1:**
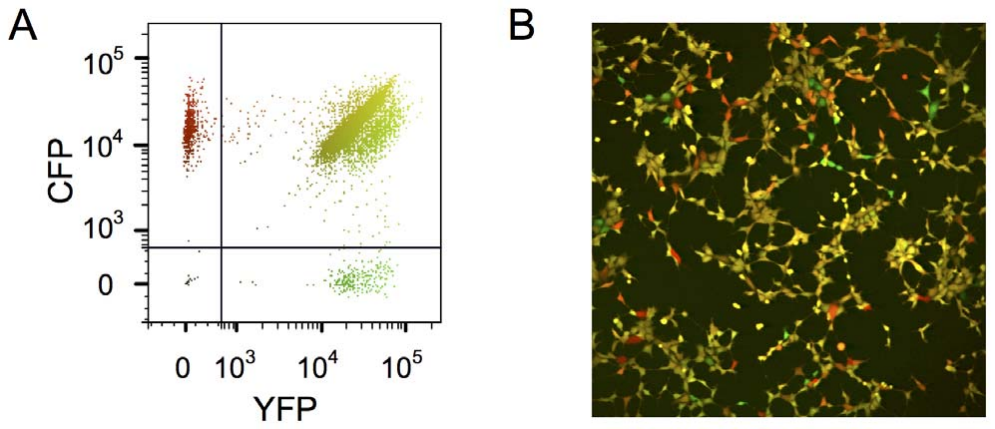
Cytometry analysis and fluorescent microscopic image of the cell clone investigated in this study. All cells carry a single copy of a CMV-YFP and a CMV-CFP transgene stably integrated in the same independent genomic locations. Cells expressing CFP are colored in red; those expressing YFP are in green. Cells expressing both reporter genes are colored with the red and green proportionally to the respective level of fluorescence. The mixture of the two colors produced different tones of yellow. Non-expressing cells are black. The same color code is used on the cytometry plot and the all the fluorescent microscopic images and movies presented in the paper. 30000 cells were analyzed by cytometry.

Gene expression is a multistep process that includes chromatin remodeling, transcription and translation, mRNA and protein degradation. All these steps are noisy and may contribute to the random variation of the protein abundance in individual cells [1], [4]. It is unclear how variations generated during these different steps influence the stable transmission of a phenotype over cell divisions. Most of the published studies used fixed time-point analysis of isogenic cell populations with the implicit assumption of ergodicity [5]. This approach allowed the identification of many different sources of variation: transcription, chromatin dynamics, unequal repartition of molecules during cell division [1], [6], [7]. However, fixed time-point studies provided no direct information about the frequency and temporal dynamics of the variation. Rapid fluctuations can result in apparently similar population patterns as slow variations. However, a population of rapidly fluctuating cells may display radically different biological properties than a population of slowly fluctuating individuals. High frequency of fluctuations may endow the cell with the capacity of rapid adaptation while low frequency promotes stable phenotype. The main steps of the gene expression process may contribute differently to the overall dynamics of the fluctuations. The identification of these contributions may reveal potentially critical stages that induce differentiation or set periods of phenotypic stability. Therefore, we analyzed the dynamics of protein abundance variation in human cells using our dual reporter gene experimental system [8]. In this model, single copies of two different reporter genes were introduced into independent genomic integration sites of cells. The two transgenes differed only by a small number of nucleotides: one encoded for cyan- the other for yellow-fluorescent protein (CFP and YFP) and both transgenes included a CMV promoter as a regulatory sequence. The use of reporter genes with a viral promoter and coding for fluorescent protein had a number of advantages in the context of our work. The fluctuations of transcription from such a promoter are expected to be independent of specific gene networks and dependent only on the variation of the overall transcriptional potential. The fluorescent proteins used are devoid of cellular functions; hence, they are not targeted by specific regulatory events, nor do they impact on the gene expression process itself. Our previous study revealed a markedly heterogeneous gene expression level within clonal populations of cells [8]. The expression of both transgenes varied considerably between the cells of the same clone and correlated only poorly with each other within the same individual cell. This suggested that the chromatin context around the transgene insertion sites is the major determinant of the gene expression level and the promoter has unexpectedly little effect on it. At the same time, the overall gene expression level distribution in each clonal population displayed remarkable stability over time independently of the average expression levels. The profile of the clones described in Neildez et al. remained essentially identical over the 5 years the cells spent in culture with subpopulations of four types (YFP+/CFP+; YFP+/CFP-; YFP-/CFP+ and YFP-/CFP-, Fig. 1) remaining in strikingly constant proportions. The population level stability can be based 1. either on the ergodic properties of the cell where rapid fluctuations in individual cells continuously restore the overall distribution; 2. or on a cellular memory mechanism that maintains the expression level of the mother cell in the daughter cells. In this case, the heterogeneity in the population is mainly generated at the early stages of clonal expansion and followed by stability in the cell lineages. In order to differentiate between the two possibilities, we investigated the temporal dynamics of the reporter gene expression variation at different time-scales: over several days, weeks or within a single cell cycle. We observed a slow and independent diversification of expression levels of CFP and YFP reporters in sub-lineages within the same clonal populations suggesting the process involves the opposing action of variance-generating and “memory” mechanisms. To clarify the relative contribution of the different stages of the gene expression process to the equilibrium between variation and memory in cell lineages, we established new cell clones with decreased mRNA and protein stability. The “memory” effect was completely lost in these clones. These observations demonstrate that in addition to chromatin-based mechanisms protein and mRNA stability are crucial to maintain a stable level of gene expression in a cell lineage.

## Results

### Parental clone characterization

Essentially all the eight clones described earlier [8] displayed a similar stable qualitative profile independently of the average expression level of each reporter gene. This suggests that all 16 chromatin integration sites impacted the long-term fluctuation profile in the same manner. Therefore, a single representative clone was chosen from the collection for detailed analysis. Although genetically identical, the cells of the clone displayed highly variable YFP and CFP fluorescence levels (Fig. 1). First, we verified if the differences of the YFP and CFP protein levels between the cells reflect the transcription of the genes. Using Q-RT-PCR, we measured the mRNA levels of the two transgenes in flow cytometry-sorted non-fluorescent, low-fluorescent and high-fluorescent cells (S1 Fig.). The relative level of transcripts correlated well with the average fluorescence intensity and no YFP and CFP transcripts were detected in non-fluorescent cells. Therefore, the protein levels correlate with the transcript levels.

A small fraction of cells of the clonal population did not express any of the transgenes (YFP-/CFP-) or only one of them (YFP-/CFP+ or YFP+/CFP-). We wondered if the silent state is determined by the epigenetic state of the corresponding promoter. We therefore analyzed the DNA methylation pattern of the CMV promoter in expressing and non-expressing cells using the bisulfite sequencing method. The promoter was found unmethylated irrespectively of the expression of the transgene (S2 Fig.), and cannot account for the transcriptional state further reinforcing the idea that the observed silencing is not due a specific regulatory mechanisms acting directly on the CMV promoter. We suspected the wider genomic context to be at the origin of transgene- and cell-dependent variation of expression levels, because the main difference between the two transgenes resided in their genomic integration sites. Unfortunately, the cloning, identification and analysis of the integration sites (S3 Fig.) provided no specific clues to understand why the transgenes were silenced in a fraction of the cells, why the expression levels varied so widely and why the two transgenes displayed independent expression levels. The YFP-coding transgene was integrated in the intron 3 of the *Glis3* gene on the Chr. 9p24.2 and the CFP-coding transgene into the intron 7 of the Wee gene (Chr15.5). We have examined the expression of the genes flanking the integration sites as well as the *Glis3* and Wee genes where the transgenes were inserted. They all are expressed at the same level in low- and high reporter gene-expressing cell fractions (S4 Fig.) suggesting that both loci are fully active in the cell type used in this study.

### Fluctuation analysis at low temporal resolution

**Figure 2:**
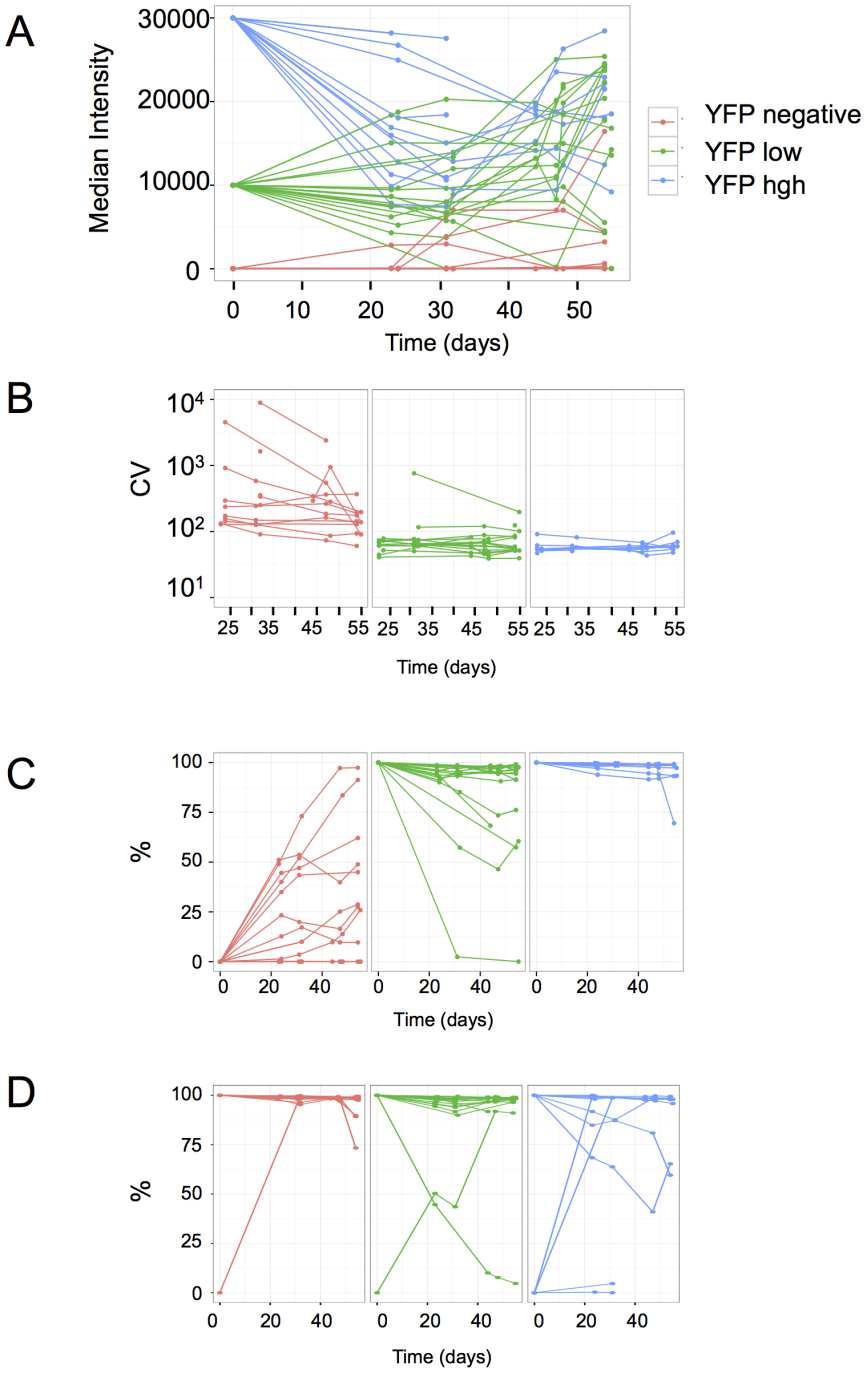
Evolution of the fluorescence level and variation in sub-clones. A. Evolution of the median YFP fluorescence level in populations derived from high- (blue line), low- (green line) and non-fluorescent (red line) founder cells. The time scale is in days after the sorting of founder cells. Note that after more than 20 days it is still possible to recognize which type of founder cell the population derived from. B. The Normalized variance (NV) of the populations originated from high- or low-fluorescent founder cells vary little over time (presented on Fig. 2A). The initially high NV in populations derived from non-fluorescent cells is the consequence of the re-expression of the YFP fluorescence in a small fraction of cells. C. The fraction of the expressing cells in the populations derived from non-fluorescent, low- or high-expressing founders. The varying proportion of expressing cells in the populations derived from non-expressing founders suggests re-expression of the reporter gene is not uniform in these clones. The populations originated from low-expressing founders contain varying proportions of cells that have silenced the reporter gene, while silencing is less frequent in the populations derived from high-expressing founders. D. The expression/silencing of the CFP-coding reporter gene is independent of the YFP-coding reporter gene as indicated by the varying fractions of CFP-expressing cells in the same subclones presented on Fig. 2A to Fig. 2C. All but one YFP negative cells were positive for CFP (left) on the day of cloning, one low YFP-expressing (middle) and four high YFP-expressing (right) clones derived from cells that did not express CFP.

Three types of subclones were derived from the original clonal population by isolating individual cells using a cell-sorter originated from cells with high YFP fluorescence, with low YFP fluorescence and from cells that did not express YFP. Overall, 12 subclones derived from high expressing cells, 18 from low expressing cells and 14 from negative cells were analyzed. CFP fluorescence was not taken into account for the cloning. The individual subpopulations derived from the isolated cells were cultured for 55 days and periodically analyzed using flow cytometry. After an initial period of 2 weeks required for the expansion of the subclones, each population was analyzed once a week by flow cytometry. Although the average YFP fluorescence in the subclones varied substantially, all of them were characterized by the general tendency of a slow relaxation to the original parental average (Fig. 2A). However, the process was strikingly slow, requiring many cell generations. Three weeks after the isolation of the founder cells, it was still possible to recognize if a population was derived from a high-, low- or non-expressing cell. The normalized variance of the fluorescence calculated as the ratio of the variance and the square of the mean (NV = σ^2^/µ^2^) remained constant all over the experiment in the high- and low-expressing subclones (Fig. 2B). This shows that the variation of the expression level is a steady and continuous process that results in the increase of the overall heterogeneity with the expansion of the population. In subclones derived from expressing cells a fraction of non-expressing cells was systematically observed indicating that complete and mitotically stable silencing of the transgene also occurred in some cells with a detectable frequency (Fig. 2C). This frequency of silencing was higher in subclones derived from low- compared to high- expressing cells. One subclone derived from a low-expressing cell became negative by the end of the experiment (Fig. 2A)

The subclones derived from initially negative YFP cells mirrored the behavior of the populations derived from expressing cells. In all but one of the analyzed 14 subclones we observed a varying proportion of YFP expressing cells (Fig. 2C). This suggests that spontaneous activation of the transgene occurred with a low but detectable frequency and that the active state was transmitted through the mitosis.

The CFP fluorescence was also analyzed in the subclones. Since they were established on the basis of YFP fluorescence of the founder cells, their original CFP fluorescence level was not known. The subsequent flow cytometry analysis of the subclones showed that the fluctuation of the CFP fluorescence also followed slow relaxation dynamics toward the original parental average (not shown) with rare switch-on and switch-off events (Fig. 2D). Despite the similar kinetics, these events did not correlate with the YFP fluorescence further demonstrating the independence of the two transgenes.

### Fluctuation analysis at high temporal resolution

**Figure 3:**
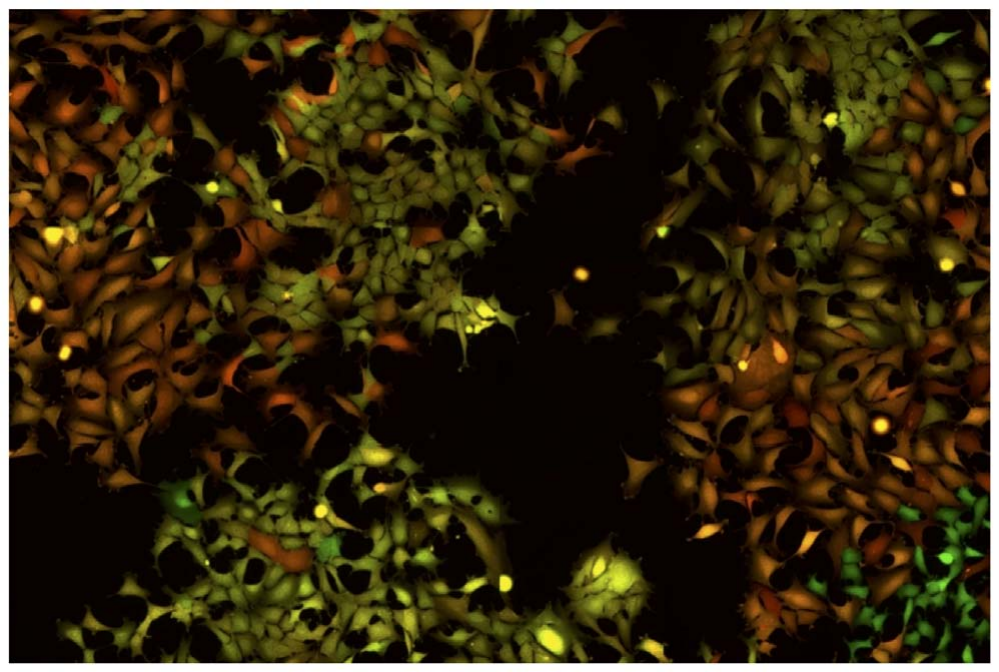
A snapshot from S1 Movie. Cells with similar level of fluorescence spread over the surface of the culture dish. The YFP and CFP fluorescence was colored artificially in red and green for better visibility. The cells expressing both YFP and CFP are colored by the proportional mixture of the two colors. It can be deduced from the movie that the close physical proximity of the cells with similar red/green ratio is the result of clonal inheritance of the expression levels of the two different fluorescent proteins.

**Figure 4:**
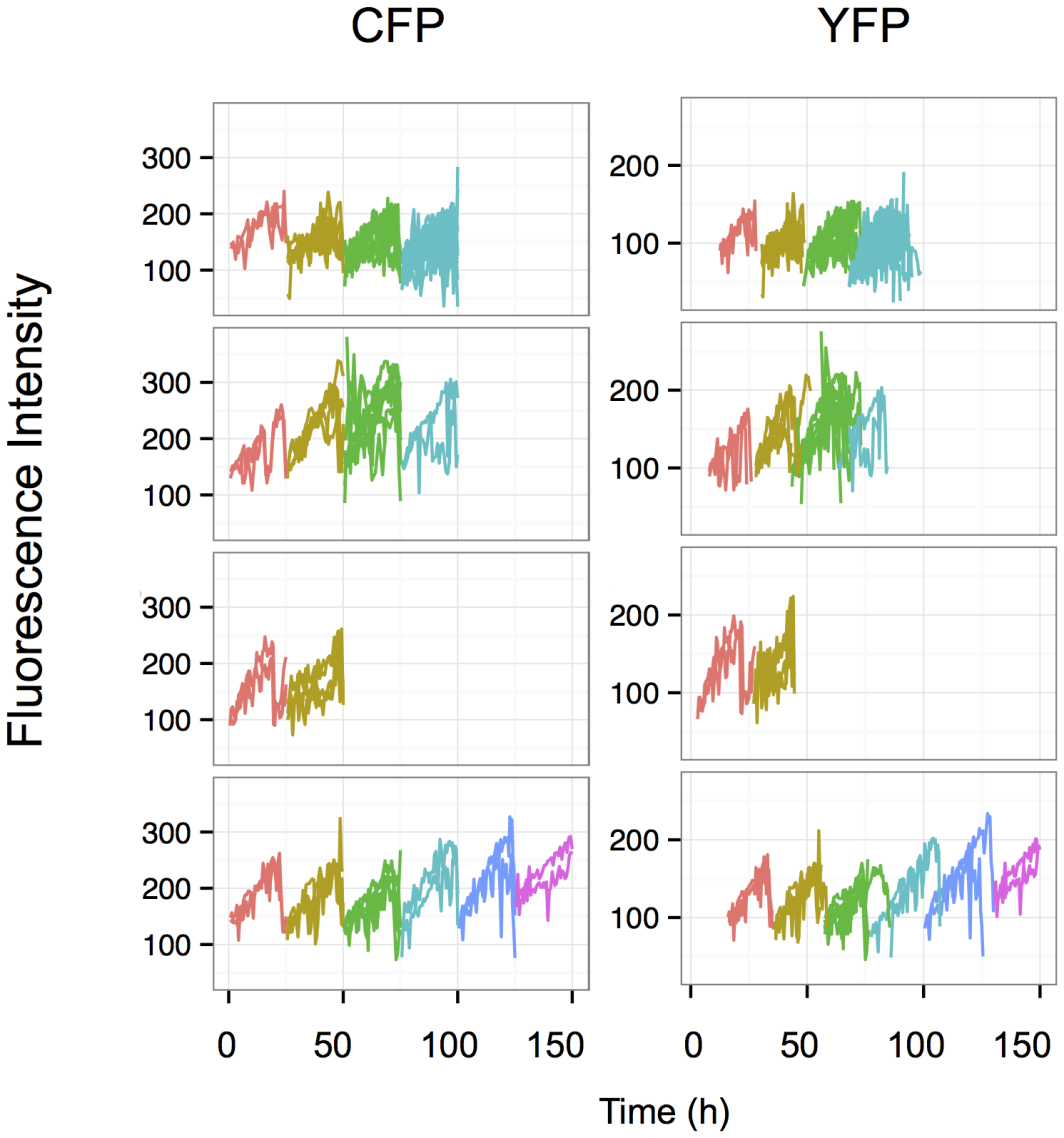
Change in the total fluorescence of individual cells and their siblings over several cell divisions. Each line represents the record of a single cell and different colors were used to indicate the different cell generations. Theoretically, the number of cells, hence the number of superposed lines is doubled at each division, but it was not always possible to follow each cell after the division. Only the cells that could be recorded over a full cycle are indicated. Note the steady increase of fluorescence in each cell during the cycle and the small variation between the daughter cells. The differences are gradually increasing over generations.

Next we sought to characterize the fluctuations at higher time resolution using time-lapse microscopy. This approach allowed us to follow individual cells and their descendants for 3 to 4 cell cycles, (up to 120–130 hours at a resolution of 1 image per 10 min). A representative movie can be found in the Supplementary files (S1 Movie) and a snapshot is shown on Fig. 3. It was particularly easy to recognize the cells belonging to the same lineage even after several divisions, because the mother cells and their siblings have essentially the same color indicating similar levels of the two fluorescent proteins. High expressing cells gave rise to high expressing lineages of cells and, reciprocally, low expressing cells produced low expressing daughter cells. This illustrates the low fluctuation of YFP and CFP fluorescence at the time scale of the time-lapse analysis. These observations are in accordance with the data obtained on the longer time-scale using cytometry and illustrate well that the cells are able to maintain the average level of the reporter proteins close to that of the cell they derived from three-four generations earlier. To better illustrate this essential point, we used an automatic image analysis to identify and track the cells and their descendants. The total and mean intensities of the YFP and CFP fluorescence in single cells were recorded. The quantitative analysis of the cells confirmed the qualitative observations. The total fluorescence of both reporter proteins increased progressively together with the cell volume during the cell cycle. During the first half of the cell cycle the increase was modest but steeply increased during the second half (Fig. 4). The total fluorescence dropped roughly to half at each division as the cell mass was divided by two. However, the cells conserved a similar average fluorescence because the cell volume also increased progressively during the cell cycle and dropped to half at division.

As shown earlier, the fluorescence level in the cells correlated with the mRNA level. However, the within-lineage stability of the fluorescence level was similar in low- and high-expressing cells, suggesting that the “memory” mechanism responsible for the transmission functioned independently of the transcription.

**Figure 5:**
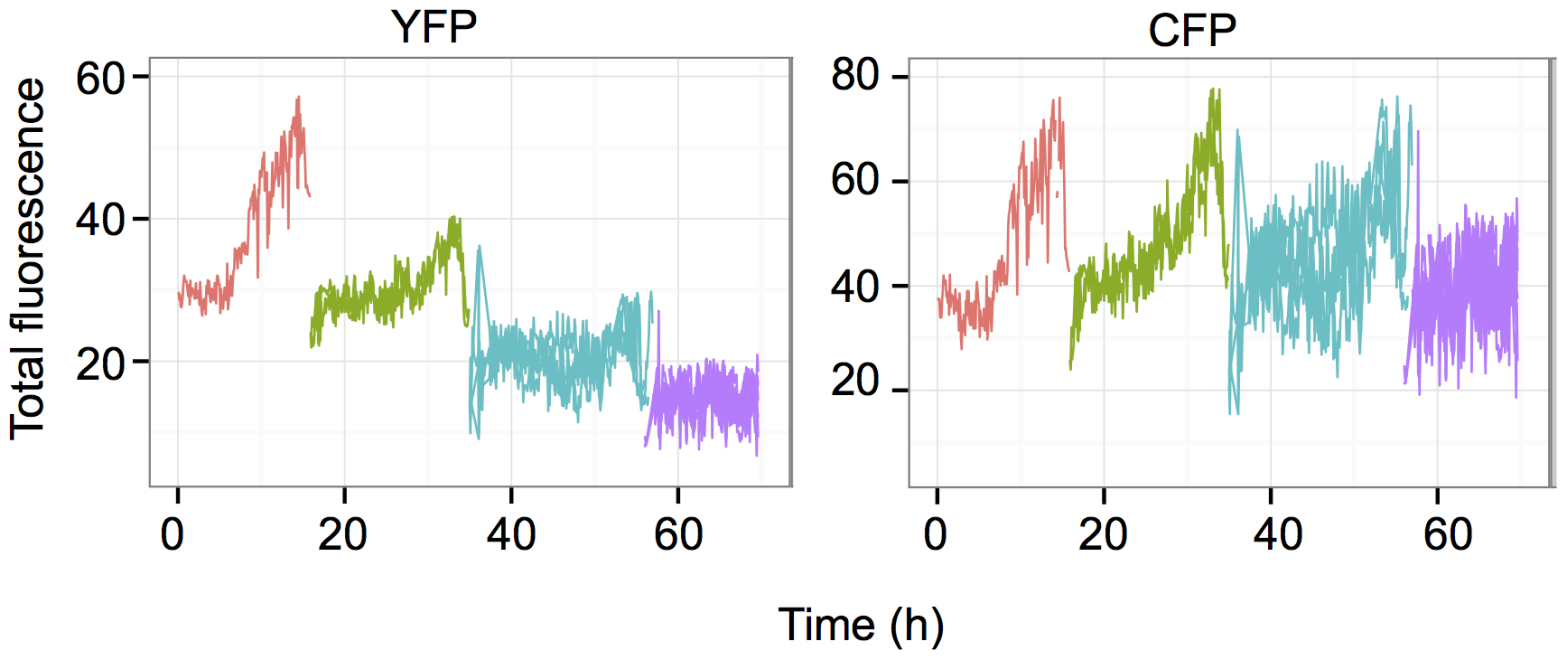
Example of a founder cell where the YFP-expressing reporter gene is silenced. The already synthesized fluorescent protein molecules are gradually diluted and degraded over the cell divisions resulting in a decrease of the total fluorescence in all sibling cells after three cycles. During this time the CFP-coding reporter gene is continuously expressed and the total fluorescence level continues to fluctuate as described before, independently of the YFP-coding gene. The color code is as on the Fig. 3.

On rare occasions, we observed cell lineages where the intensity of one of the two fluorescent proteins decreased continuously. One representative cell lineage is shown on the Fig. 5. The mother cell displayed typical behavior, but the YFP fluorescence started to decrease simultaneously in the daughter cells and further decreased in the subsequent generations. This continuous decrease suggests that the transcriptional silencing of the reporter gene presumably occurred in the mother cell and was transmitted stably over the division and the already synthetized mRNA-s and fluorescent proteins became diluted over several cell generations resulting in a gradual decrease. The ratio of such silencing events was only 1–2%. The CFP level however, remained stable in the same cell lineage.

### Computer simulations

The observations both on short- and long time-scales show slow reporter gene expression level variation over several cell generations. This global kinetics of the fluorescence fluctuations reflects the overall outcome of the gene expression process that includes production and degradation of the YFP and CFP proteins. However, the impact of the various steps on the overall kinetics may differ substantially. In order to discriminate between the relative impact of the different steps on the overall clonal stability of the gene expression level in our experimental system and to formulate experimentally testable hypotheses we performed computer simulations using a ‘random-telegraph model’ [9] modified to include regular cell replication events during which the mRNAs and the proteins quantities are equally divided between the mother and daughter cells. More precisely, our model simulates the levels of proteins in an individual cell as a function of chromatin opening/closing rates, frequency of transcriptional bursts during the open chromatin periods, rates of mRNA and protein production and degradation and frequency of division events.

The main conclusion emerging from the simulations concerns the strong within-lineage correlation, or “memory effect” of the expression levels. The long mRNA- and protein half-lives appear as key parameters for the maintenance of the expression level over several cell generations.

**Figure 6:**
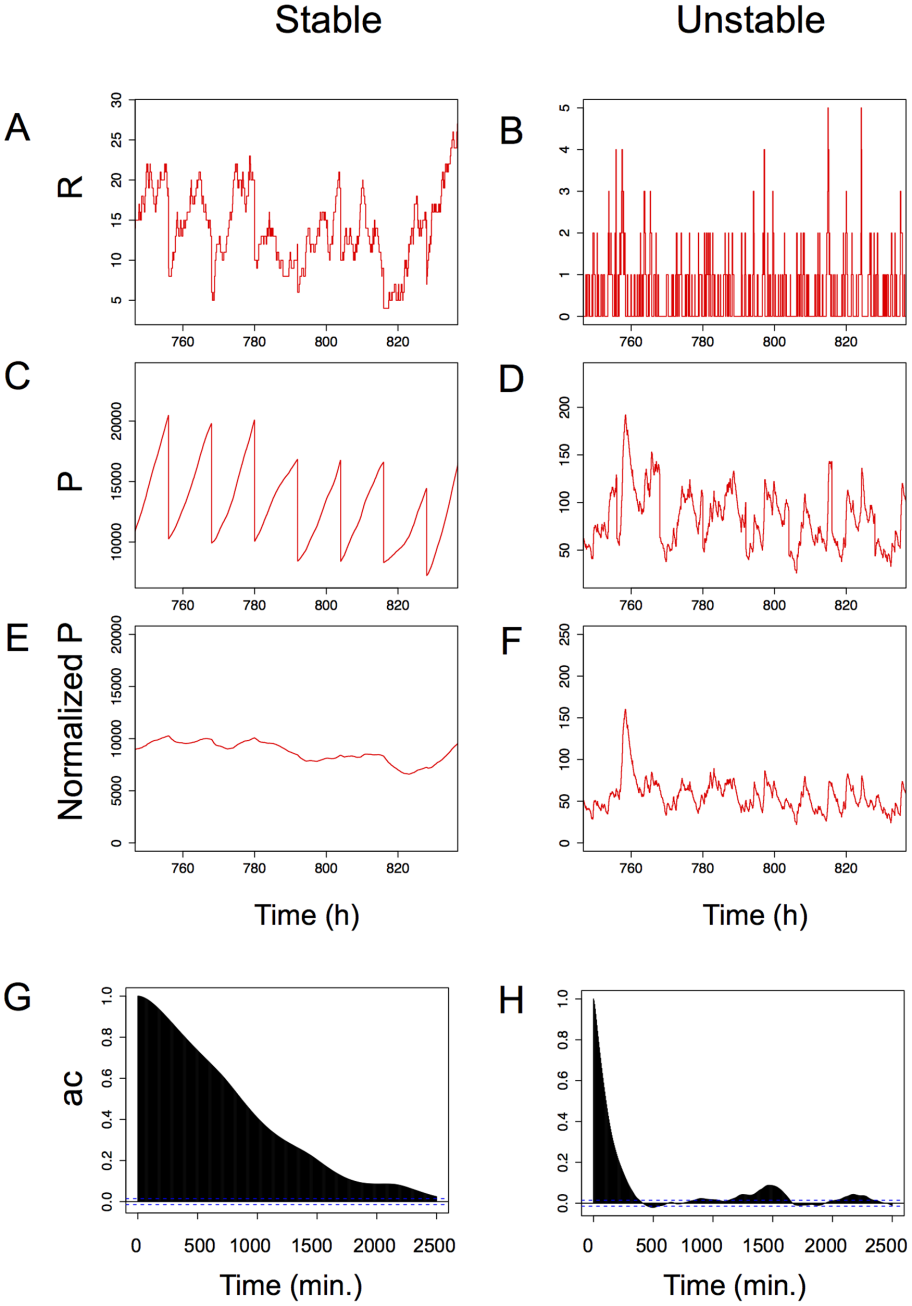
Computer simulation of the effects of protein stability on the evolution of the total fluorescence in a clonal population. Here we consider a period during which the chromatin state is constantly open. The results obtained with long half-lived mRNA and proteins are shown on the left side and those with short half-lived proteins and mRNA on the right side. Panels A (resp. B) through G (resp. F) represent changes in number of molecules in a single cell and its daughter cells during 7 divisions for stable molecules (resp. unstable molecules). All times are in minutes. Note the steady increase of the Protein number during the cell cycles and the rapid decrease during the divisions (panel C) and the irregular fluctuations for a similar simulation with unstable molecules (panel D). Panels E and F represent the number of protein normalized by a hypothetical volume increasing linearly from 1 to 2 during the cell cycle (that is to say the mean fluorescence level). Stable molecules lead to low stochasticity (panel E, NV = 0.017) during the open chromatin state. Unstable molecules reveal a highly stochastic behavior (panel F, NV = 0.11) even though chromatin remains stably open. This difference is further exemplified on panels G and H that show the autocorrelation function calculated on the normalized protein concentration on the basis of very long simulated data (120 divisions). Note the rapid loss of autocorrelation in cells with unstable mRNA and proteins (panel G) compared to cells with stable mRNA and proteins (panel H). The detailed description of the model and the parameters can be found in S1 File.

When the half-life of the protein was reduced in the model, the memory effect disappeared and the autocorrelation of the fluorescence levels over time decreased drastically (Fig. 6). This observation can be interpreted as follows: The amount of fluorescent protein in a cell is determined by the balance between synthesis, degradation and regular halving during cell division. The synthesis rate depends on the slow chromatin opening/closing kinetics and the faster transcription/translation reactions. When degradation is slow, the cell may reach the end of the cycle and divide before the protein level reaches the equilibrium. As a result, the protein level in the cell (reflected by the total fluorescence in the experiments) is essentially limited by the regular dilutions due to cell division. Hence, the slow variations of the protein level predominantly reflect the fluctuations of the chromatin on-off dynamics that may result either in the halting of the synthesis process during possibly long-lasting closed- or in open and transcriptionally potentially active chromatin states. When the mRNA and protein degradation rates are high, the equilibrium level is low and can be reached before the division event. The rapid variations of this level now reflect the bursty fluctuations of the transcription/translation.

We have tested experimentally these predictions by the construction of new clones with short-live mRNA and proteins (see bellow).

The computer simulations also revealed that the existence of stable subpopulations with one or both reporter genes inactivated over several generations, this being the primary consequence of the low chromatin on-off switch rates (S5 Fig.). Rare switches lead to long periods of closed chromatin when transcription is impossible and allow the decrease of fluorescence by gradual dilution over several cell cycles as it was observed on the time-lapse records (Fig. 5). This prediction on the rare chromatin switches is also experimentally testable (see the next section).

### The chromatin on/off rates are low

**Figure 7:**
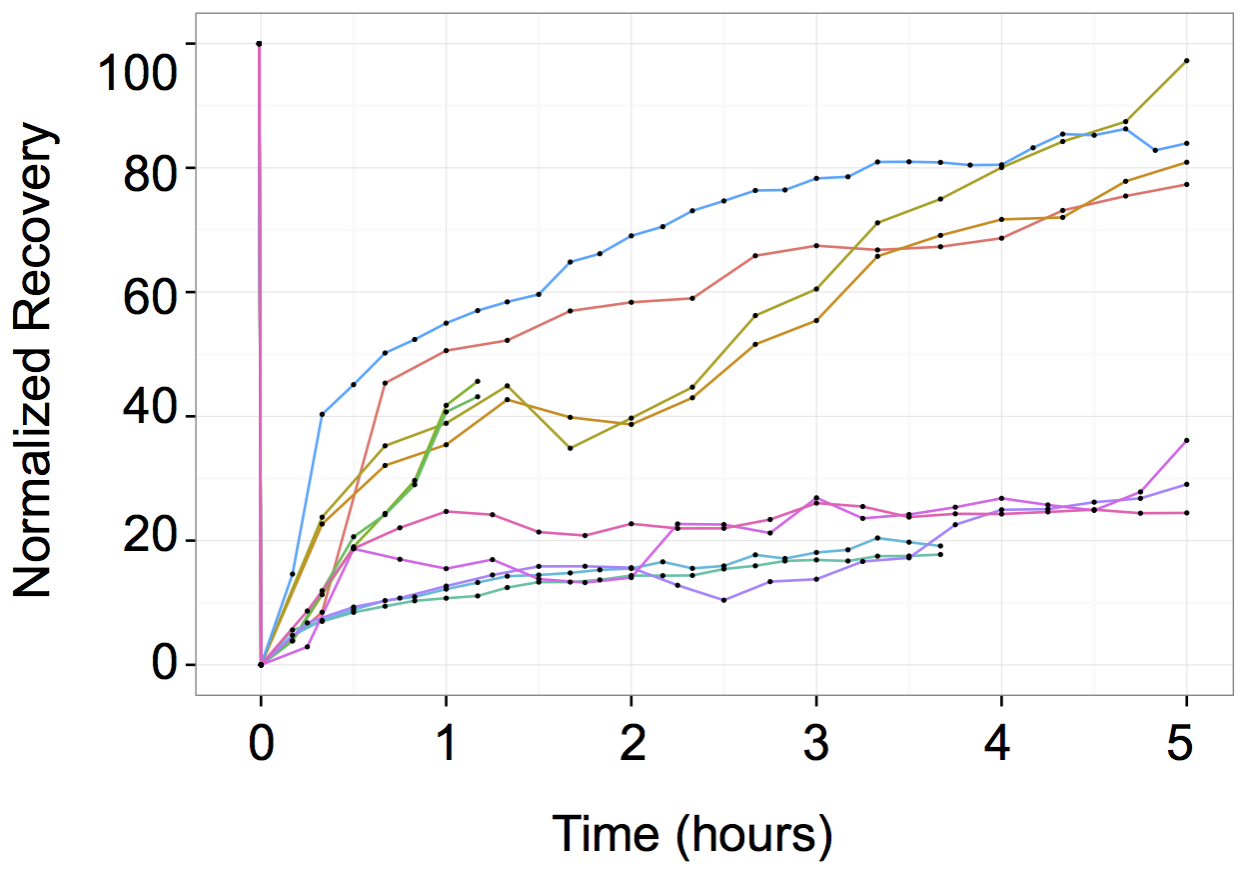
Fluorence Recovery After Photobelaching (FRAP) analysis of the YFP fluorescence in whole cells. Each line represents the fluorescence of a single cell (n = 11). Note the two distinct kinetics of fluorescence recovery. Approximately half of the cells recovered fluorescence immediately after photobleaching suggesting active YFP synthesis. The fluorescence in the second group of cells remained as low as in control cells treated with transcriptional or translational inhibitors.

In order to explore the predictions of the model, we set up two types of experiments. First, we sought to measure the frequency of transcriptional bursts. To do this, we performed whole-cell photobleaching studies. The experimental strategy was founded on the idea that rapid increase of fluorescence is expected following a transcriptional burst during the phase of protein synthesis. Previous time-lapse measurements failed to detect the increase presumably because the increment of fluorescence was too small to be easily differentiated from the already high fluorescence of the highly stable YFP and CFP expressed by the cells. To overcome this difficulty, we reduced the fluorescence of the YFP to 20% of the initial level by photobleaching. The intensity of CFP was kept unchanged as control and used to normalize the YFP intensity. The YFP fluorescence of randomly selected cells was bleached and the cells were monitored for 5 hours. The normalized YFP/CFP results are shown on Fig. 7. Although the low number of the cells analyzed did not allow a precise statistical estimation, it was clear from these results that approximately only the half of the cells in the population started to recover the YFP fluorescence immediately after the bleach indicating active transcription and translation of the YFP reporter gene. The other half behaved exactly as the control cells (S6 Fig.) treated with an inhibitor of transcription (5,6-dichloro-1-β-D-ribofuranosylbenzimidazole, DRB) or translation (cycloheximide). This observation suggests that neither transcription occurred in these cells during the 5 h period of observation nor mRNA molecules were available for translation in these cells. These observations confirm the model prediction on the low chromatin on/off switch rate.

### The long mRNA and protein half-lives determine cellular memory

Next, we sought to clarify the effect of the long mRNA and protein half-lives. To do this, we created new reporter gene-expressing cell lines. We modified the yellow and cyan gene constructs in a way to decrease the reporter half-lives. We used a destabilizing AU-rich element (ARE) [10] and the PEST sequence of the mouse ornithine decarboxylase [11] to reduce the mRNA and protein half-lives respectively. The experimental measurements indicated that the half-life of the mRNA decreased to 1.5 h for YFP and 1.9 h for CFP, less than the half of the corresponding half-life in the cells used in the first part of this study. In the case of the proteins, the introduction of the “PEST” sequence had an even stronger effect: the half-life of the YFP decreased from the average 43 h to 6 h30 and that of CFP from 29 h30 to 5 h40.

**Figure 8:**
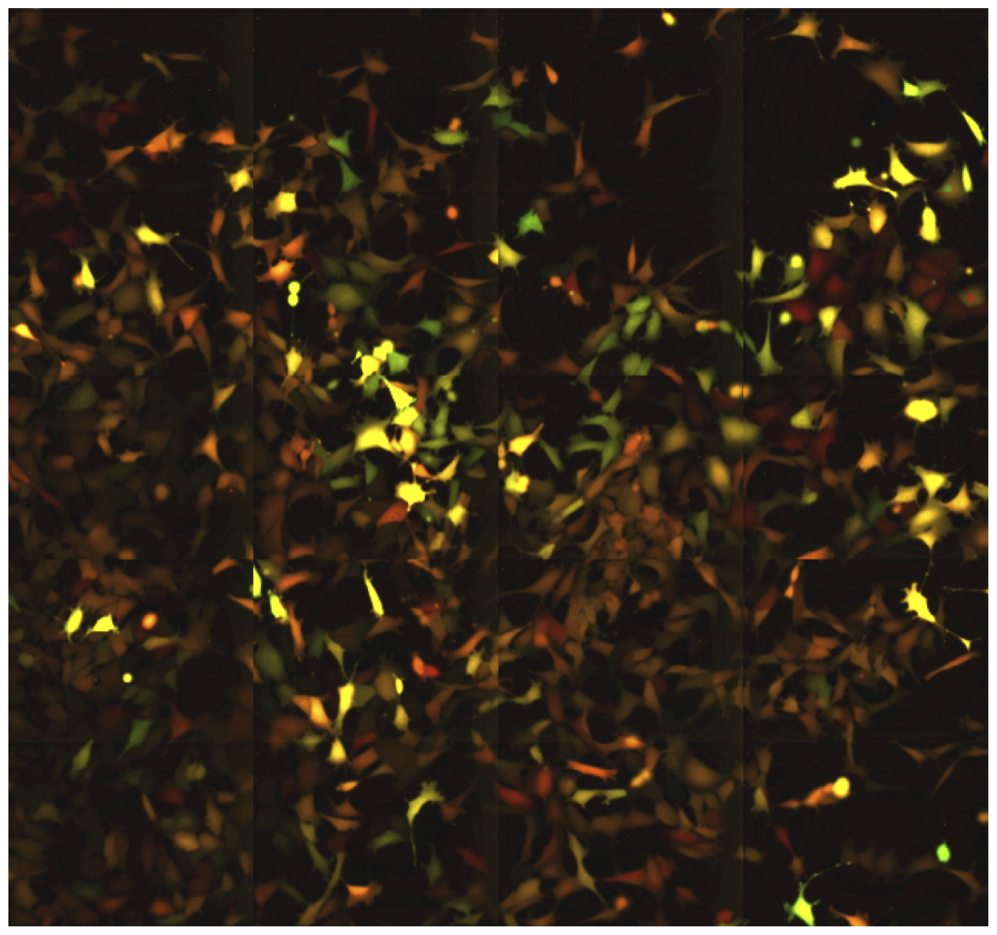
A snapshot from S2 Movie. The video shows cells expressing varying levels of fluorescence proteins spreading over the surface of the culture dish in a clonal population expressing the short half-lived fluorescent proteins. The YFP and CFP fluorescence was colored artificially in red and green for better visibility. It can be seen on the S2 Movie that there is no correlation between the fluorescence level of mother and daughter cells and there is no clear clustering of the cells with similar YFP/CFP ratio.

**Figure 9:**
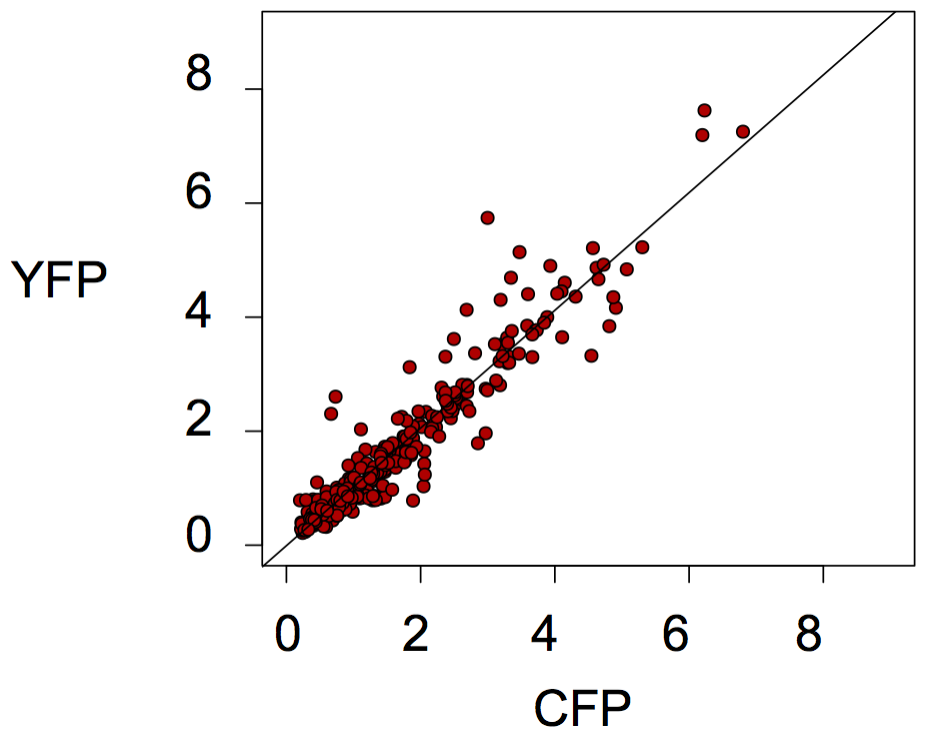
Correlation of the half-life of the YFP and CFP fluorescent proteins. The time required to reach 50% autocorrelation in the cells of a clone expressing short half-lived YFP and CFP fluorescent proteins. Autocorrelation was determined by measuring the total fluorescence of the cells on a time-lapse movie. Note the short time required for each protein (the consequence of the rapid degradation of the mRNA and proteins) and the good correlation between the two fluorescence proteins (presumably the consequence of the shared degrading mechanisms and the independence from the transcriptional activity). Each point indicates a single cell from the same clone. Overall, 128 cells were analyzed. The time scale is indicated in hours.

Many independent clones have been established. All of them displayed qualitatively similar fluctuating gene expression suggesting that the reporter gene's integration site did not impact significantly the temporal dynamics of fluctuations. Two representative clones with single insertion sites for both transgenes were analyzed in detail. As expected on the basis of the short half-life of the mRNA-s and proteins, the average level of YFP and CFP fluorescence measured by flow cytometry was found 100 times lower than the levels observed in cell clones with the stable proteins. Despite the large difference in the expression levels, the snapshot of the expression profile was similar in the two types of clones with cells that expressed only one fluorescent protein or that were negative for both. Time-lapse video microscopy analysis revealed that the fluorescence level of both reporter proteins now varied substantially and with a high frequency (Fig. 8 and S2 Movie). The period of the changes was shorter than the cell cycle, so that the same cell could change fluorescence between two divisions. A simple visual inspection was sufficient to conclude that no lineage-specific correlation of the fluorescence can be seen in these clones. On the basis of the time-lapse records we quantified the fluorescence fluctuations in about 120 cells from the two clones and calculated the autocorrelation functions for YFP and CFP in each cell and the time τ_1/2_ for the autocorrelation to drop to 50%. Although the cell-to-cell differences are important, both for YFP and CFP τ_1/2_ was less than 2 h in the majority of cells, illustrating the loss of the “cell memory” observed previously (Fig. 9). The half-life of the two proteins correlated well within the same cell suggesting that they are degraded through the same pathway (Fig. 9). Therefore, the rapid fluctuations of the fluorescence were made possible by the short lifetime of the mRNA-s and the corresponding fluorescent proteins and presumably directly reflect the fluctuations in the transcriptional activity of the genes.

## Discussion

In the present study we have analyzed the temporal stability of gene expression in clonal populations of cells with two independent reporter genes having identical promoters but coding for different fluorescent proteins. As described earlier, the transcriptional activity of the two copies of the identical promoter was significantly different even in the same individual cells. This resulted in a substantial population level phenotypic heterogeneity of the reporter gene expression levels within the same clone [8]. Different clones differed by the average expression level of the population demonstrating the role of the reporter gene integration site in determining the average rate of transcription. However, the temporal behavior of these clones was similar. In the present study, we analyzed one representative clone in detail. Contrary to our expectations, the gene expression levels in individual cells fluctuated very slowly. Daughter cells displayed almost identical levels of yellow and cyan mean fluorescence than the maternal cell resulting in easily recognizable lineages of cells with similar expression levels within the same population (Fig. 4 and S1 Movie). When individual cells were sub-cloned from the same clonal population, it took several weeks and many cell divisions for the initially different fluorescence levels in the subclones to gradually regress to the average of the initial population (Fig. 2A). We are dealing therefore with two opposite processes; the first is a “memory” process that tends to maintain the same expression level of the proteins over many cell generations while the second process introduces variation and gradually erases the memory expression level. Our observations clearly show that in our model mRNA and protein stability are key players in the memory process. The average half-life of the CFP and YFP proteins exceeded the length of two cell cycles. The protein molecules synthesized in the maternal cell have great chances to be still present in granddaughter cells. As a result, the high protein stability filters the rapid fluctuations generated by the noisy transcription bursts. The dominating slow fluctuations of the fluorescence level reflect the chromatin dynamics that determines the transcriptional potential of the reporter gene. When cell clones coding for short half-life mRNA and proteins, but transcribed from identical promoters were established the memory effect was lost. We observed rapid fluctuations of the fluorescence levels in these cells with a characteristic time scale of a few hours. These rapid variations presumably reflect the high transcription burst frequency of the CMV promoter that was undetectable in the clones with the stable reporter proteins. Therefore, these observations demonstrate that by buffering the rapid fluctuations due to transcriptional bursts the high stability of the messenger and the protein product contributes to the conservation of the gene expression level over many cell division. This role is traditionally attributed to chromatin related epigenetic mechanisms.

Indeed, chromatin-based mechanisms are known to transmit the transcription state of a gene over cell divisions and this is obviously the case in our cell model also. In the cells with the stable YFP and CFP proteins, the mRNA levels correlate with the corresponding fluorescence levels indicating that the overall synthesis rate is determinant for the protein abundance. YFP and CFP fluorescence levels may differ substantially within the same cell. The difference in transcription between the two reporter genes is not due to the promoter, because an identical promoter drives the two genes. It is the chromatin structure at the integration site that determines if transcription can occur [7]. Chromatin structure also accounts for the transmission of the silent state during mitosis. This is particularly evident in low expressing cells. Cells derived from a low expressing founder remain low expressing for many generations suggesting that the low rate of mRNA synthesis was transmitted through the cell division. One reporter gene can even become silenced while the other reporter gene remains fully active. This total independence of the two genes driven by identical promoters can only be explained by the dominant influence of the chromatin at the integration site on the transcription potential. However, we observed that the memory of the overall gene product level is lost in cell clones with short half-lived mRNA and proteins in spite of the fact that the same CMV promoter was used as in the long half-lived mRNA/protein-expressing clones. The difference in the memory is therefore correlated with the stability of the gene products and not the transcriptional activity.

Our observations extend the concept of cellular memory by showing that the conservation of the stable phenotype in a cellular lineage may largely depend on the very slow turnover of the fluorescent proteins. The volume and the mass of the maternal cell increase about twofold by the end of the cell cycle and this material is roughly halved during the division. In order to maintain the same level of a protein (in terms of number of molecules/volume) over the cell cycle and in the daughter cells after division, the total amount of the protein has to double by the end of the cycle. Hence, to achieve the stability of the phenotype in proliferating cells the protein synthesis rate has to exceed the degradation rate. This can be achieved even at low transcriptional burst frequency is the stability of the gene product is high. This is exactly what we see, but only in the long half-lived mRNA/protein-expressing cells (Fig. 3); the total YFP and CFP fluorescence reflecting the total number of fluorescent protein molecules of each species increases during the cell cycle and falls at the moment of division.

It is very important to remind that stability is not an intrinsic property of the protein molecule itself. As a substrate of degrading enzyme(s), it is determined by the affinity (measured by the Michaelis constant) and the frequency of interactions between the protein and the degrading enzyme, but also by the frequency and affinity of interactions with partner proteins that may protect it from degradation.

Stable cell phenotype in the cell lineage means that the concentrations of key proteins that bring about it remain stable over the cell divisions. It is interesting from this viewpoint that proteins of similar stability were found to belong to the same functional category [12]. This observation can be interpreted by considering that the proteins of the same functional network frequently interact physically with each other. These interactions are essential both for the stability of the network as a whole but may account for the stability of the individual proteins also [13]. It appears therefore compelling that mutual stabilization of the proteins by frequent interactions may be an important factor of phenotypic stability and cellular memory. If high stability of the proteins contributes to the maintenance of the phenotype in the cells, the opposite may also be true; it may hamper the cell's capacity to respond quickly in case of environmental stress. Therefore, protein stability may be an important target of regulation; a rapid degradation of functional proteins could be a first step to a rapid phenotypic conversion.

Although the CFP and YFP fluorescence levels remained essentially stable over several cell generations under normal growth conditions, gradual drift was observed. Independently of the starting level in the founder cell, the average level and the distribution of the fluorescence in the population derived from it gradually approximated that of the original clonal population the founder cell was isolated from. The relaxation to the average is a hallmark of stochastic fluctuations. It is likely, that it results from random variations at all stages, because chromatin dynamics, gene transcription, protein production and degradation are all noisy processes. This was particularly evident in the case of the repressed chromatin state. The spontaneous stable reactivation resulting in a mosaic expression in a clonal population was the manifestation of the chromatin noise (Fig. 2C). Another factor that could contribute to the generation of heterogeneity was the length of the cell cycle [14]. Cells with a long cell cycle synthesized more proteins and gave rise to daughter cells with higher fluorescence than those that divided early. As a result, after a few cell divisions, the initially small differences accumulated and contributed substantially to the heterogeneity in the population.

Overall, the comparison of cell clones expressing high or low-stability reporter proteins raises a number of important points. The extent of variation is similar in both types of clones; these cells visit all possible expression level states from the lowest (non-expressing) to the highest (maximal expressing). However, the typical time scale of these variations is different in the two types of clones suggesting that they depend on different mechanisms. In the high stability protein-expressing clones the expression level and the fluctuations essentially reflect the effect of chromatin dynamics around the transgene integrations sites. Integration sites are unique and independent; as a corollary the characteristic expression levels and fluctuations differ even within the same cells. The memory effect due to the chromatin and the long half-life of the mRNA-s and proteins results in stable subpopulations with different expression patterns even within populations of isogenic cells. Clearly, in our system at least, the chromatin fluctuations are slow with rare switches between the repressed close and the transcriptionally competent open chromatin states, leading to stable subpopulations that repress none, one or both of the two reporter genes. Similar situation has been reported in a different cell model [7]. By contrast, in the low stability protein expressing cells the fluctuations essentially reflect the noise arising during the initiation of transcription, mRNA synthesis and transport processes. The high stability of the reporter protein in the first type of clones successfully buffered these fluctuations, but the short half-life of the mRNA-s and proteins reveal them.

The study of the temporal dynamics of the reporter gene expression variation at different time-scales provides a proof of principle that the epigenetic memory of phenotypic stability in a cell lineage emerges from the joint action of the process of gene transcription/protein- synthesis/degradation/cell division. Protein stability is crucial for the capacity of the cell to maintain a stable level of gene expression and raises an important possibility that it could be a target for efficient regulation when rapid change in the gene expression level is required. Although this conjecture has been discussed recently, the impact it may have on the epigenetic inheritance of cellular phenotypes during cell divisions remains underestimated [15]. Indeed, our observations suggest that the stability of the cellular mRNA and proteins confers the capacity to a cell to conserve a stable gene expression level and transmit it over multiple generations even if transcription and translation are highly fluctuating. In addition, reducing short-term fluctuations through high stability of the molecules can be considered as simple way of transcription noise reduction at a low energy cost. Indeed, it takes less energy for the cell to maintain the constant level of a protein by not degrading the molecules already present than continuously re-synthesizing them.

## Materials and Methods

### Cell model and cell culture conditions

The cells were routinely cultured in Dulbecco's modified Eagle's medium (DMEM, cat. 31966, GIBCO) containing 10% Foetal Calf Serum (FCS) (cat. 10270-106, GIBCO) and antibiotics (Penicillin 100U/ml and Streptomycin 100 µg/ml, GIBCO) at 37°c in a humid atmosphere and 5% CO_2_.

The cell clones used were originally described in [8]. They were derived from 911 cells, a Human Embryonic Retinoblastoma cell line [16] transfected with a plasmid containing the enhanced cyan fluorescent protein (CFP) reporter gene under the control of the cytomegalovirus (CMV) promoter. A population of stable CFP expressing cells was isolated using a cell sorter and transfected with a second construct containing the enhanced yellow fluorescent protein (YFP) reporter gene under the control of an identical CMV promoter. Double cyan/yellow fluorescent cells were isolated using a cell sorter and expanded. Two of the eight clones described in Neildez et al. containing a single integrated copy of each transgene were selected for further analysis in this study.

The integration sites of both transgenes in each clone were identified by splinkerette PCR [17].

### Flow cytometry analysis and cell sorting

Cells were detached from the culture dish using trypsin/EDTA (Gibco), mixed with DMEM containing 10% FCS and centrifuged after sifting with a 40 µm cell strainer to remove cell clumps (Becton Dickinson). Cell pellet was suspended in 1x PBS (Gibco) containing 2% FCS. A dead cell staining was done with Propidium iodide at 2 µg/ml final concentration (Sigma) and cells either analysed or used for sorting.

Flow cytometry acquisition and analysis were performed using a LSR II cytometer and the FACS DIVA software (Becton Dickinson). Typically, CFP and YFP were excited at 405 nm and 488 nm respectively and fluorescence collected using a 525/50 and a 530/30 filter, respectively. In order to compare results from one day to another, calibration beads were used each day. In each experiment, 10^4^ PI negative cells were analysed.

Cell sorting was performed using a MoFlo cell sorter (Cytomation). CFP and YFP proteins were excited using a 445 nm and a 488 nm (100 mW) excitation source respectively. Fluorescence was collected using a 485/25 nm filter and a 575/25 nm filter. Cells were collected in a 96 wells culture plate (Becton Dickinson) containing 100 µl of complete medium and incubated in standard conditions. Two days later, 50 µl of complete medium were added in each well and the presence of cells checked using an epifluorescence microscope. When the cell density had reached 80%, cells were transferred to a 12 wells, then a 6 wells plate and finally to a 25 cm^2^ dish where they were routinely diluted and expanded.

### Time Lapse imaging

Cells were seeded in a 35 mm culture dish (cat. P35G-0-14-C, MatTek corporation) at low density (10^3^ cells/cm^2^). The next day, the dish was set in a CO_2_-regulated chamber under a confocal microscope (Zeiss LSM 510 Meta) for imaging. The microscope whole system is enclosed in a thermo-regulated structure. The dish was incubated 1 hour under the microscope in order to stabilize the temperature of the whole system before acquisition. CFP and YFP fluorescence were acquired using a 10x dry objective, at 457 nm and 514 nm excitation wavelength respectively and fluorescence collected with a 480–520 IR and 535–590 IR emission filter respectively. Time delay between consecutive frames was set to 10 minutes and the image resolution to 1024*1024 using an 8 bits pixels depth.

### Whole cell photo-bleaching

The culture dish with the cells was set up as mentioned for time-lapse experiments. An image of YFP and CFP fluorescence was acquired before the photo-bleaching with a 40x immersion objective. Then, a Region Of Interest (ROI) was drawn around each cell to be bleached at zoom 2x and the bleach performed. YFP fluorescence was bleached at 2x magnification with 20 pulses of a full power 514 nm wavelength laser (100 mW, Argon). A time-lapse acquisition was then performed at zoom 1x with a time delay of 10 for 6 hours. In each experiment, we monitored bleached cells but also control cells that were not illuminated.

Bleaching settings were optimized in regard to laser power, number of pulses and magnification. Typically, with these settings, 80% of YFP bleaching is achieved and cell viability is not significantly affected. When indicated, cells were treated with the transcription inhibitor DRB (5 µg/ml) for 30 min before photobleaching (SIGMA) or the translation inhibitor cycloheximide (10 µM) 1 hour before photobleaching (SIGMA).

### Image Analysis and Data processing

Time-lapse images were automatically analysed using the freely available CellProfiler software [18]. The image analysis pipeline includes image intensity normalization, cell segmentation, cell tracking and quantification of morphological, spatial and fluorescence features. Movies and mosaics were edited manually with ImageJ.

The statistical analysis and graphical representations of flow cytometry and image analysis data's were performed using the “R” software [19] updated with the ggplot2 library [20].

## Supporting Information

S1 Fig **Correlation between the fluorescence level of reporter genes and the abundances of their mRNA-s.** The cytometry profiles of the high- and low-CFP and YFP fluorescent subclones are shown on the left panel. The normalized mRNA levels determined in the same cells using quantitative RT-PCR are shown on the right panel.
(TIFF)

S2 Fig **Bisulfite methylation analysis of the CMV promoter of the transgenes.** The methylation profile in cell subpopulations expressing both transgenes (right panel) and negative for both (left panel) were investigated. Each row of circles indicates a sequenced clone. Open circles indicate unmethylated CpG-s and black circles methylated sites.
(TIFF)

S3 Fig **Localization of the genomic integration sites of the transgenes.**
(TIFF)

S4 Fig **Quantitative RT-PCR analysis of the expression of genes at- and flanking the integration sites of the CFP- and YFP coding transgenes.** The levels of expression of these genes are similar in low- (grey bars) and high (black bars) reporter gene-expressing cell fractions as indicated on a normalized scale.
(TIFF)

S5 Fig **Computer simulation of the effects of protein stability on the evolution of the total fluorescence.** The simulated period of time was longer than 60 days. The results obtained with long half-lived mRNA and proteins are shown on the left side (panels A, C, E and G) and those with short half-lived proteins and mRNA on the right side (panels B, D, F and H). A and B show the state of the chromatin (open or closed). C through F panels represent changes in number of molecules (RNA and Protein) in a single cell and its daughter cells during 65 divisions (times in days). G and H represent the number of protein normalized by a hypothetical volume increasing linearly from 1 to 2 (that is to say the mean fluorescence level). Note that the global variance (NV = 1.26 for panel G and NV = 1.39 for panel H) is driven by the chromatin state.
(TIFF)

S6 Fig **Effect of transcription (left) and translation (right) inhibition on the recovery of fluorescence after whole cell photobleaching.** The number of cells examined in each experiment is indicated by “n”. Note the lack of recovery in both cases except a small and rapid initial increase due presumably to the termination of ongoing reactions or partial fluorescence recovery of some bleached molecules.
(TIFF)

S1 File **Supporting Materials and Methods.**
(DOCX)

S1 Movie **Time-lapse movie of the cell clone expressing the stable YFP and CFP proteins.** The YFP and CFP fluorescence was colored artificially in red and green for better visibility. The cells expressing both YFP and CFP are colored by the proportional mixture of the two colors. Note that the mother cells and their siblings have essentially the same color indicating similar levels of the two fluorescent proteins. One image was recorded every 10 minutes for five days.
(MOV)

S2 Movie **Time-lapse movie of the cell clone expressing the unstable YFP and CFP proteins.** The YFP and CFP fluorescence was colored artificially in red and green for better visibility. The cells expressing both YFP and CFP are colored by the proportional mixture of the two colors. Note that the cells change fluorescence intensity during a single cell cycle. As a result, individual cell lineages cannot be tracked on the basis of the fluorescent protein expression level. One image was recorded every 20 minutes foe 3 days.
(MOV)
