# Supplementary figures and images for: Stochastic Fluctuations and Distributed Control of Gene Expression Impact Cellular Memory
Source: PLoS One. 2014 Dec 22;9(12):e115574. doi: 10.1371/journal.pone.0115574 (PMC4274012; doi:10.1371/journal.pone.0115574)

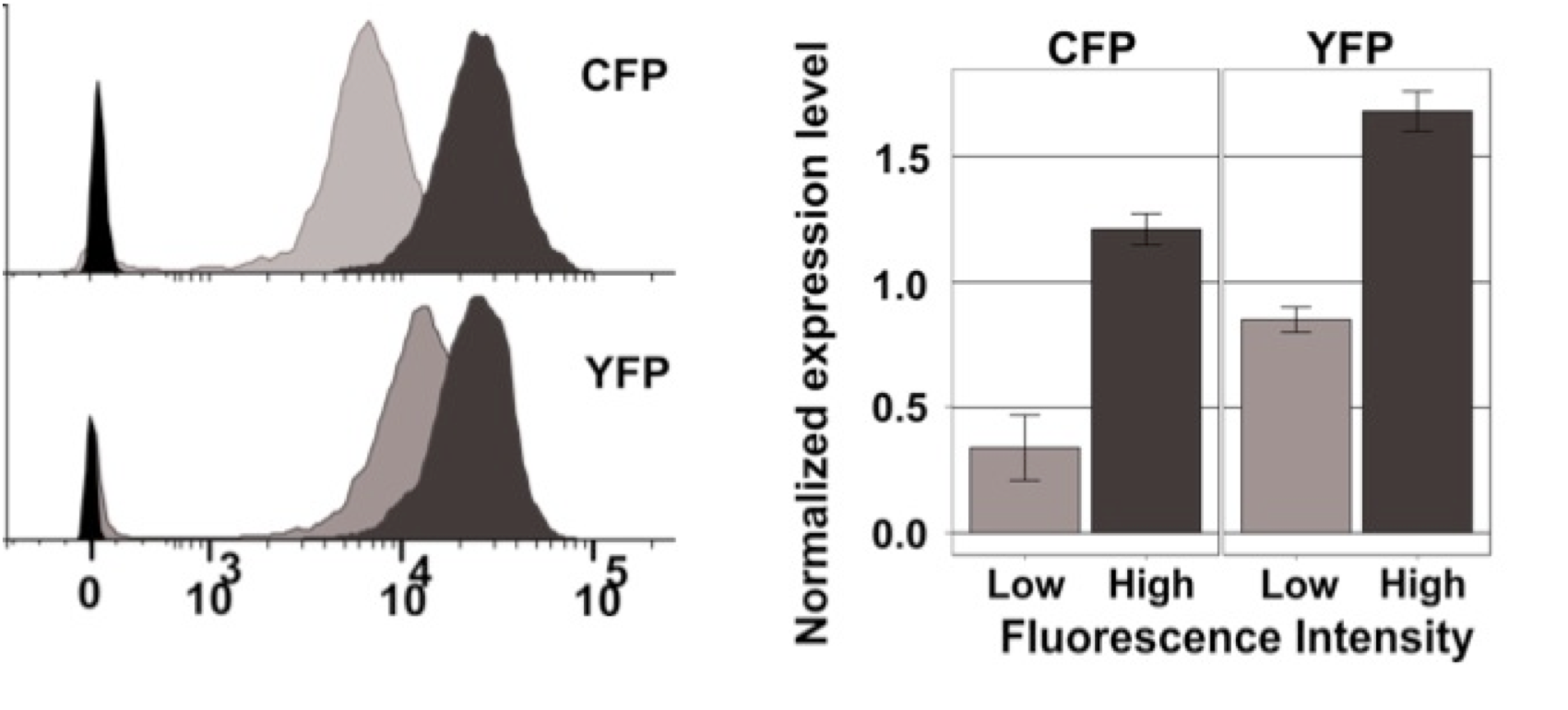

Supplement: S1 Fig — Correlation between the fluorescence level of reporter genes and the abundances of their mRNA-s. The cytometry profiles of the high- and low-CFP and YFP fluorescent subclones are shown on the left panel. The normalized mRNA levels determined in the same cells using quantitative RT-PCR are shown on the right panel. (TIFF) [file pone.0115574.s001.tiff]

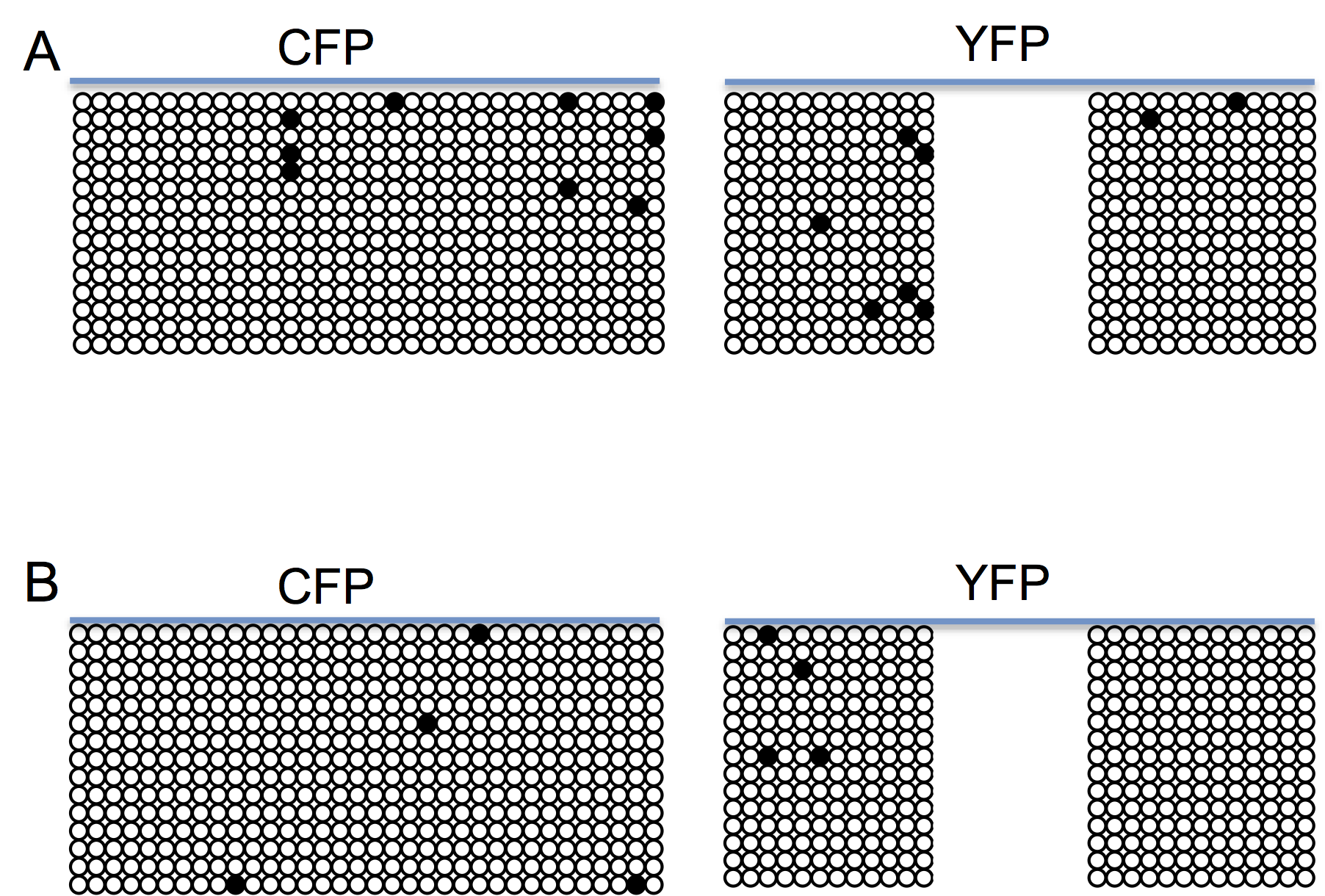

Supplement: S2 Fig — Bisulfite methylation analysis of the CMV promoter of the transgenes. The methylation profile in cell subpopulations expressing both transgenes (right panel) and negative for both (left panel) were investigated. Each row of circles indicates a sequenced clone. Open circles indicate unmethylated CpG-s and black circles methylated sites. (TIFF) [file pone.0115574.s002.tiff]

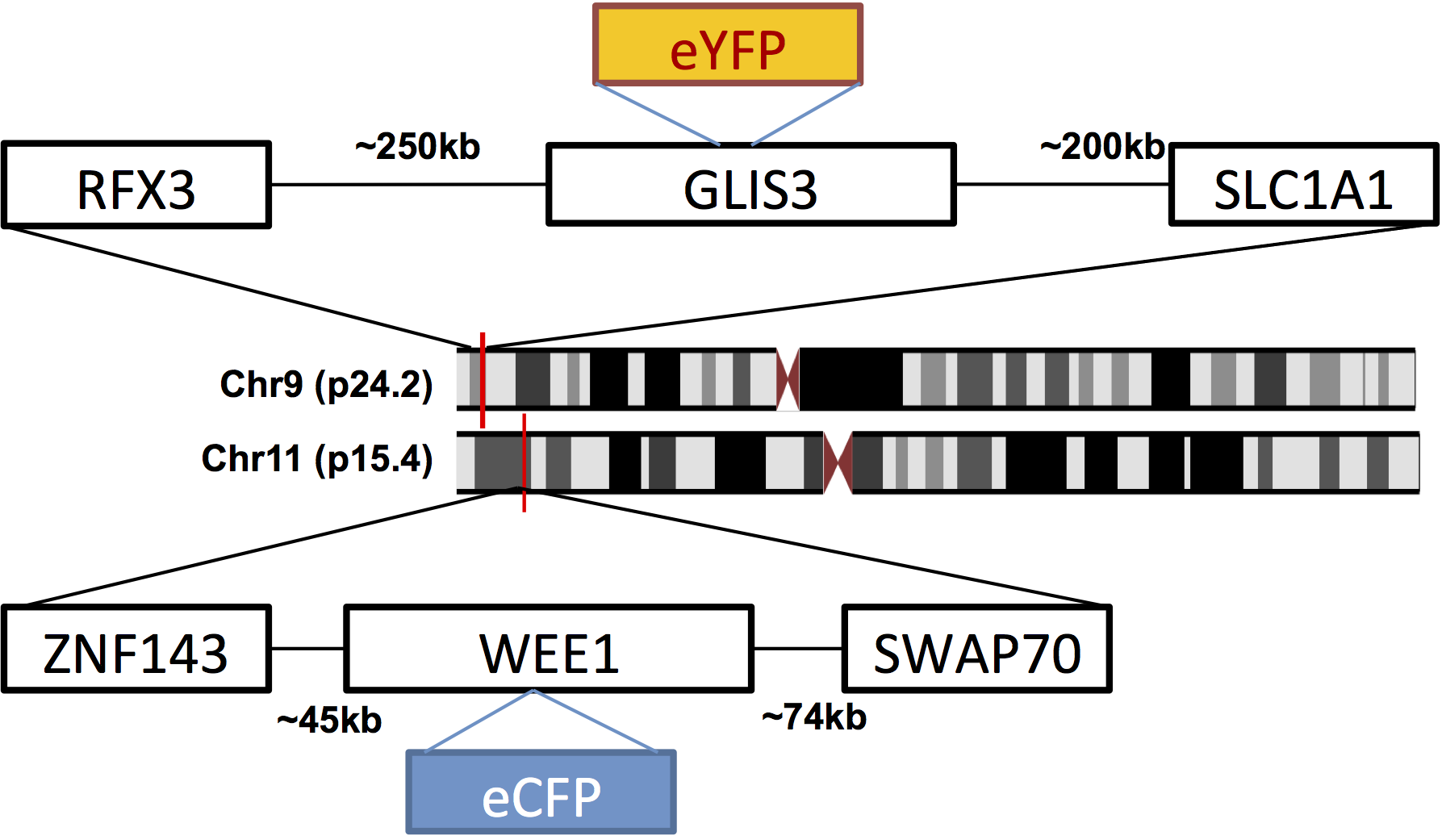

Supplement: S3 Fig — Localization of the genomic integration sites of the transgenes. (TIFF) [file pone.0115574.s003.tiff]

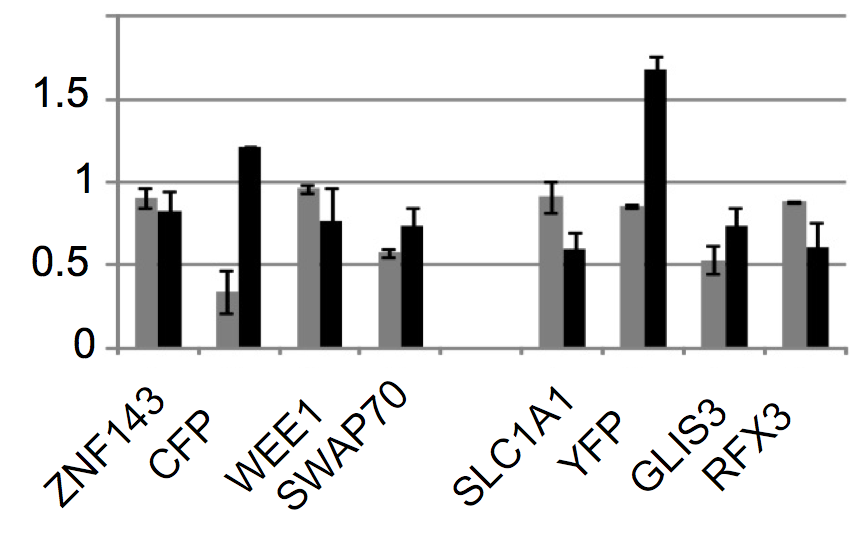

Supplement: S4 Fig — Quantitative RT-PCR analysis of the expression of genes at- and flanking the integration sites of the CFP- and YFP coding transgenes. The levels of expression of these genes are similar in low- (grey bars) and high (black bars) reporter gene-expressing cell fractions as indicated on a normalized scale. (TIFF) [file pone.0115574.s004.tiff]

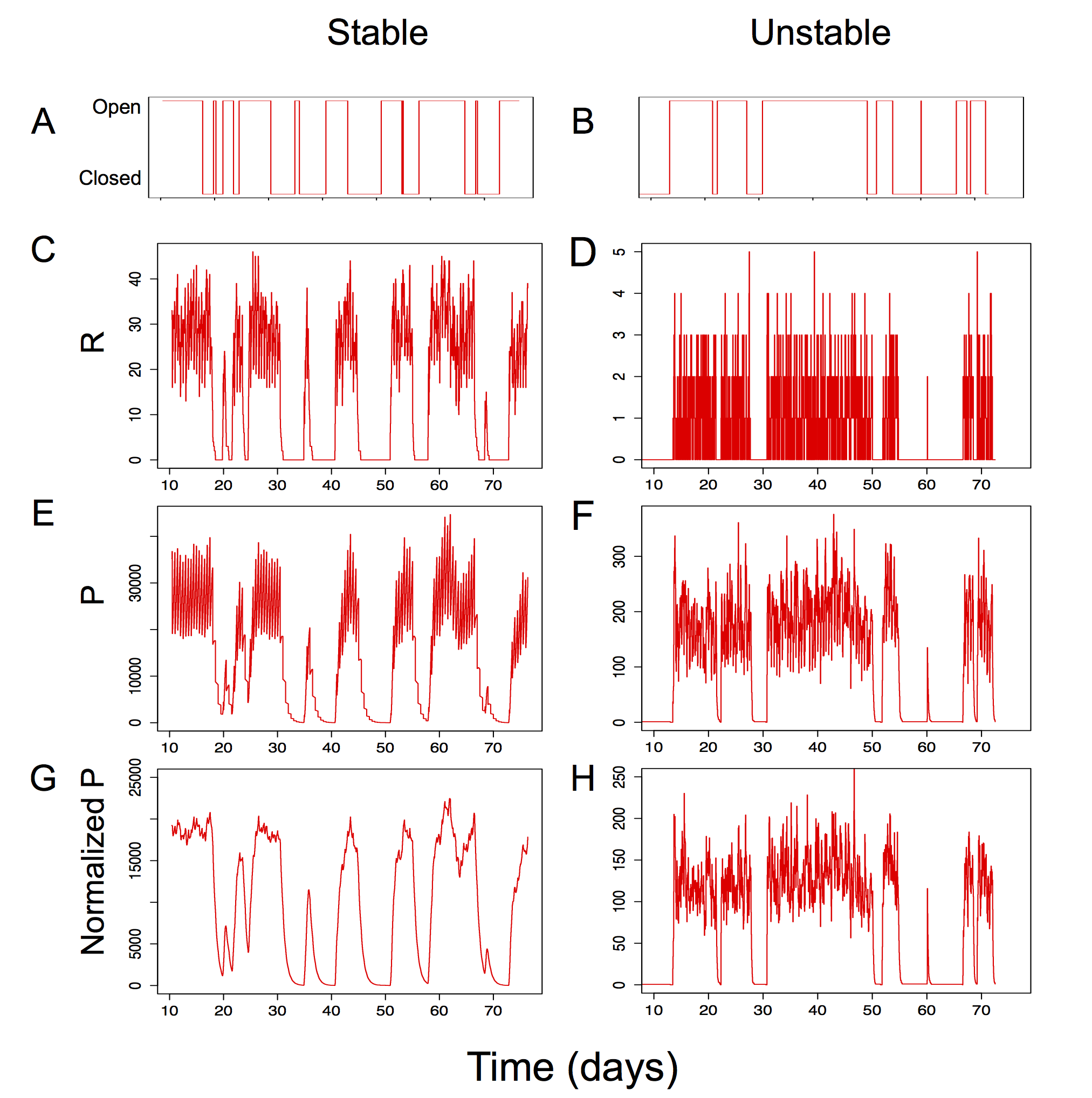

Supplement: S5 Fig — Computer simulation of the effects of protein stability on the evolution of the total fluorescence. The simulated period of time was longer than 60 days. The results obtained with long half-lived mRNA and proteins are shown on the left side (panels A, C, E and G) and those with short half-lived proteins and mRNA on the right side (panels B, D, F and H). A and B show the state of the chromatin (open or closed). C through F panels represent changes in number of molecules (RNA and Protein) in a single cell and its daughter cells during 65 divisions (times in days). G and H represent the number of protein normalized by a hypothetical volume increasing linearly from 1 to 2 (that is to say the mean fluorescence level). Note that the global variance (NV = 1.26 for panel G and NV = 1.39 for panel H) is driven by the chromatin state. (TIFF) [file pone.0115574.s005.tiff]

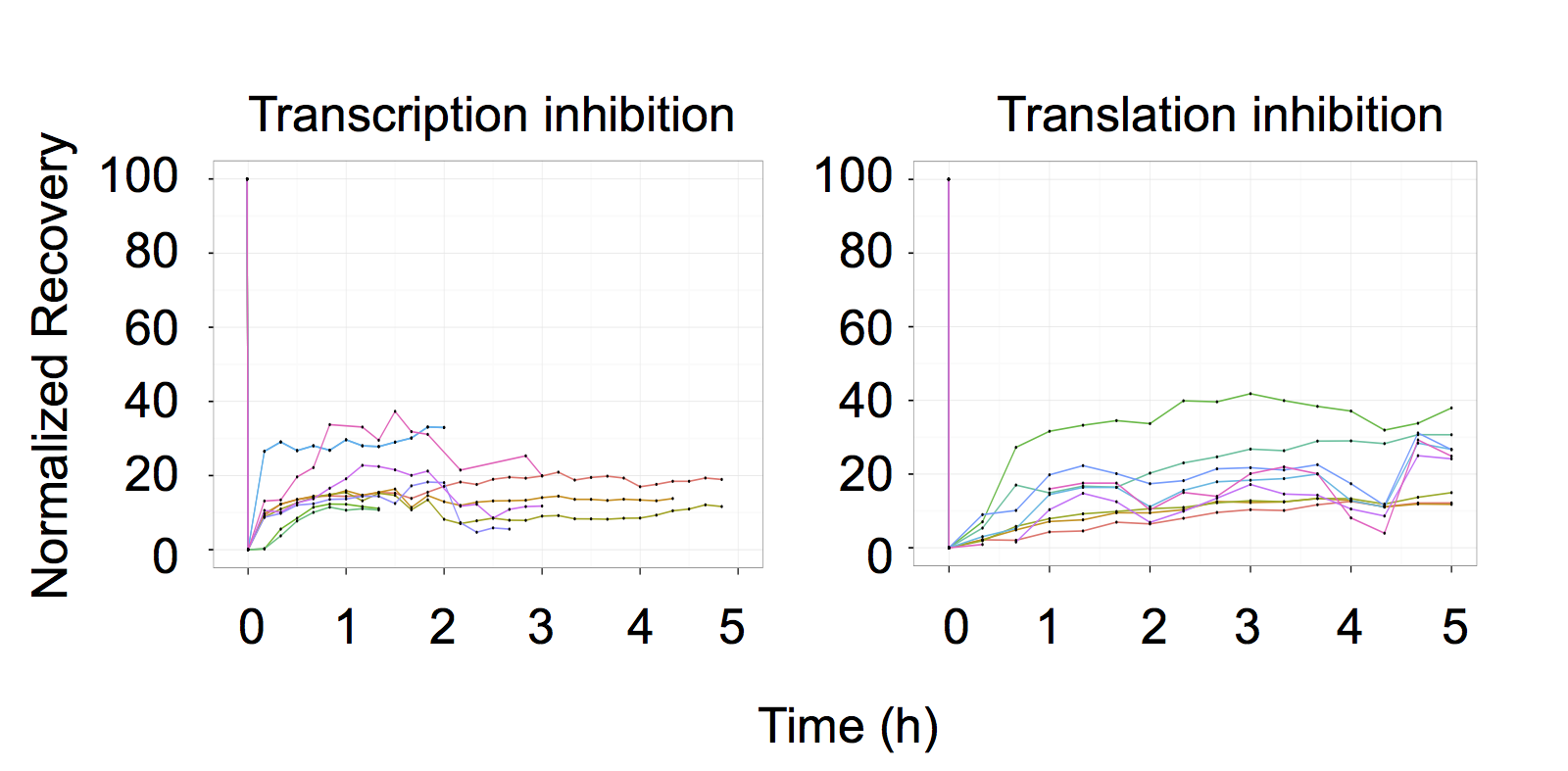

Supplement: S6 Fig — Effect of transcription (left) and translation (right) inhibition on the recovery of fluorescence after whole cell photobleaching. The number of cells examined in each experiment is indicated by “n”. Note the lack of recovery in both cases except a small and rapid initial increase due presumably to the termination of ongoing reactions or partial fluorescence recovery of some bleached molecules. (TIFF) [file pone.0115574.s006.tiff]
