# Supplementary material for: Stochastic Fluctuations and Distributed Control of Gene Expression Impact Cellular Memory
Source: PLoS One. 2014 Dec 22;9(12):e115574. doi: 10.1371/journal.pone.0115574 (PMC4274012; doi:10.1371/journal.pone.0115574)
Supplement: S1 File — Supporting Materials and Methods. (DOCX) [file pone.0115574.s007.docx]

**Supporting Information**

**Supporting Material and methods**

### *qRT-PCR Analysis*

Total RNA was extracted using the RNeasy plus mini kit (Qiagen) from 5x10^5^ cells and DNAse treated according to the manufacturer indications (Turbot DNA-free kit, Ambion). Reverse transcription was done using the Superscript First strand synthesis System (Invitrogen) from 1µg of total purified RNA and using random primers. “No RT “controls, water control and a standard curve for each template were done. A real time TaqMan PCR was done to quantify both CFP / YFP expression and TFIID was used as an internal control (Taqman Gene Expression MasterMix). Thermal conditions were 95°c for 15 min and 40 cycles using 95°c for 1min and 60°c for 1 min.

Primers: YFP forward: CTCGTGACCACCTTCGGCT; YFP reverse: TCCTGGACGTAGCCTTCGG;

CFP forward: CGTGACCACCCTGACCTGG; CFP reverse: TCCTGGACGTAGCCTTCGG;

hTFIID forward: GAGAGCCACGAACCACGG; hTFIID reverse: ACATCACAGCTCCCCACCAT;

CFP/YFP Taqman probe: CGACCACATGAA GCAG;

hTFIID taqman probe: TGTGCACAGGAGCCAAGAGTGAAGA

The expression of the genes around the integration sites was analysed by conventional RT-PCR. We used the following primers:

hZNF143forward :ACTCTGTTGCTATGGTTACTGC; hZNF143reverse :AGGTCTGGTTTCTGTGGTTAC

hSWAP forward: TCCTTTCCCATAACCTGTGC; hSWAP reverse: CACAGAGGGTCCAACACATC

hRFX3 forward: TCGAGCACTTCCAGAGTTTG; hRFX3 reverse: TGGAGTAGAGGGAGAATAGCG

hSLC1A1 forward: GTCCTGACTGGGCTTGCAA; hSLC1A1 reverse: CAACGGGTAACACGAATCG.

hWEE1 forward : ACAAGTTGAAGAGGGCGATAG ; hWEE1 reverse : CCTGTCTGATTTCATGCCATTG

hGLIS3 forward : AGCCAACCTGAATATACCTCC ; hGLIS3 reverse : AGATCAAGGCCATTCTGAGAG

### *Analysis of the protein and mRNA half-lives.*

Half-lives of mRNA and proteins were estimated by monitoring the mRNA and fluorescence decay after transcription and translation inhibition in live cells. Cells were routinely cultured in complete medium supplemented with either 5µg.ml^-1^ DRB or 50µg.ml^-1^ cycloheximide (CHX) for 0 to 5 hours. mRNA quantification was performed by qRTPCR targeting CFP and YFP mRNA. The number of cells from whom the RNA was extracted was used for the normalization. Protein decay was estimated by monitoring CFP and YFP fluorescence by flow cytometry after different delays of treatment. Both mRNA and proteins decay following the respective treatments was fitted to an exponential decrease to determine mRNA and proteins half-lives.

### *Integration site cloning by Splinkeret PCR*

Integration sites of both CFP and YFP in clonal populations were identified using the protocol, adapted from ([Uren et al, 2009](#_ENREF_17)). Genomic DNA was extracted using genomic tips (Qiagen,) with Genomic DNA buffer set (QIAGEN). DNA was then completely digested with the restriction enzyme CviQI (Fermentas). This enzyme cuts the CFP and YFP constructs integrated in the genome only at a single site. The digested DNA was purified using the PCR purification kit (QIAGEN). After digestion, DNA fragments displays a 5’ TA –overhang. Splinkerette PCR adaptators (“Splink long” and “Splink short”) were annealed by progressive cooling after a complete thermal denaturation. The double stranded adaptor containing a cohesive end that match the restriction enzyme digestion profile was then ligated to the digested DNA with DNA ligase (Invitrogen). 35 rounds of amplifications were performed using a primer located in the adaptor (Splink1) and multiple reverse primers located in the transgene. A nested PCR was then done using a primer located in the adaptor (Splink2) and another one located in the construct. The PCR product mix was ligated in a pGEM-easy vector (Promega) according to manufacturer protocol. Cloned inserts were sequenced using SP6 and T7 primers on a 3130xl sequencer (Applied) and the genomic integration sites were identified using BLAST.

The following primers were used: Splink 1: CGAAGAGTAACCGTTGCTAGGAGAGACC; Splink 2: GAAGAGTAACCGTTGCTAGGAGAGACCGTGGCTGAATGAGACTGGTGTCGACACTAGTGG; Splink long: GAAGAGTAACCGTTGCTAGGAGAGACCGTGGCTGAATGAGACTGGTGTCGACACTAGTGG; Splink short: TACCACTAGTGTCGACACCAGTCTCTAATTTTTTTTTTCAAAAAAA.

### *CMV promoter methylation*

Genomic DNA was prepared from CFP/YFP expressing and double negative subpopulations purified by cell sorter from the original heterogeneous clonal population. The DNA was subjected to bisulfite conversion using the EpiTect Bisulfite kit (Qiagene) according to the manufacturers instructions. A fragment spanning the promoter and a part of the protein coding sequence was amplified by semi-nested PCR using the following primers: CMV forward 01: ATTTACGGGGATTTTTAA; CMV forward 02: GGGATTTTTAAGTTTTTAT; CMV-GFP reverse: ATAAACTTCAAAATCAACTT. The fragments were purified, cloned and sequenced. YFP- and CFP-derived clones were identified on the basis of their sequence. Those derived from the YFP transgene contained 8 CpG-s less.

*Model description*

The two-states model of gene expression represents the chromatin activity as an ‘on-off’ process specified through the transition rates *kon* and *koff* (respectively representing the ‘off-on’ transition and the ‘on-off’ transition). mRNA and protein dynamics are based on production/degradation models where the production of mRNA was allowed only in the ‘on’ state (open chromatin) but completely forbidden in the ‘off’ state (closed chromatin).

The model formulation is therefore described by 6 reactions as follows:

1. $Closed\left( G=0 \right)\underset{\to}{kon}Open(G=1)$

2. $Open\left( G=1 \right)\underset{\to}{koff}Closed(G=0)$

3. $0\underset{\to}{kr}R if G=1$

4. *R*$\underset{\to}{dr}0$

5. $R\underset{\to}{kp}P$

6. $P\underset{\to}{dp}0$

Reaction 1 and 2 correspond to the state transition of the chromatin between an open state (*G* = 1) and a closed state (*G* = 0) where, *kon* is the closed-to-open transition rate, *koff* is the open-to-closed transition rate. Reactions 3 and 4 correspond to the RNA production and degradation where *R* is the number of mRNAs, *kr* is the mRNA production rate (when chromatin is open), and *dr* is the mRNA degradation rate. Reactions 5 and 6 correspond to the translation and protein degradation where *P* is the number of proteins, *kp* is the protein production rate (per mRNA) and *dp* is the protein degradation rate. The stochastic model consists of these 6 events that occur randomly at exponentially-distributed time intervals with the respective propensity functions:

1. $kon \times(1-G)$

2. $koff \times G$

3. $kr \times G$

4. $dr \times R$

5. $kp \times R$

6. $dp \times P$

In addition, the model includes the division of the cell that occurs every 12 hours with reduction of the number of molecules *R* and *P* by 2 and conservation of the chromatin state *G*.

Simulation of the model

The model can be simulated using an SSA, which is an exact continuous-time algorithm that enables simulation of chemical-reaction systems. Each simulation represents one of the possible realizations of the system from a specified initial state and for a given kinetic parameter set (these parameters being here considered as probabilities). Each realization depends on a pseudo-random generator, and different realizations (that is, simulations of different cells issued from the same clone) can be computed by simply initializing this random generator with different seeds. The implementation of the two-state model in the SSA enables simulation of the entire system dynamics and visualization of the course of chromatin state, gene transcription, number of mRNAs, mRNA translation, number of proteins and, ultimately fluorescence, in a virtual single cell.

By simulating the dynamics of cell states for a long time number of such ‘artificial cells’, we were able to compute evolution of the number of RNA and Proteins for a given parameter set. In order to compute the autocorrelation function and the Normalized Variance (NV) of the mean fluorescence level, we normalized the number of proteins by a hypothetical volume increasing linearly from 1 to 2 during the cell cycle.

For each set of parameters, we simulated the virtual cell for 120,000 minutes (a sufficiently long period to ensure that all cells were at a steady state, the concentration values being initialized at 1 molecule per cell).

Parameters for the degradation reactions (*dr* and *dp*) were *dr* = 0.003 min^-1^ and *dp* = 0.0001 min^-1^ for stable mRNA and protein. These values were inspired by the experimental measured half-life of the stable YFP and CFP that is close to 48 hours and is close to 3 hours for the stabilized CFP and YFP mRNAs. The values used for the unstable mRNA and protein were *dr* = 0.1 min^-1^ and *dp* = 0.005 min^-1^.

We explored parameters ranges for *kr* from 6 to 0.01 min^-1^ and for *kp* from 1 to 0.001 min^-1^. For values of *kr* higher than 0.05 and for values of *kp* higher than 0.1 and for a condition where the chromatin state is always open (*koff* = 0), we obtained systematically lower NVs (between 0.001 and 0.028) and higher autocorrelation for the simulations using parameters for unstable mRNA and Protein compared to NVs for unstable mRNA and Protein. We used the following values for the figure 6 and supplementary figure S5: *kr* = 0.111 min^-1^ and *kp* = 1 min^-1^.

A wide range of parameters for the probabilities of the transitions between the close and the open chromatin states were also explored. We were interested in the probabilities that produced a ratio of negative/positive populations of 1/5 and individual cell states maintained for a long period of time. We knew from the time-lapse observations that fluorescent and non-fluorescent cells tend to keep their state for several days and several cell cycles. The following representative values that produced effects similar to the experimental observations were selected for supplementary figure S5: *kon* = 0.002 min^-1^ and *koff* = 0.001 min^-1^

The mathematical model was implemented using R software and a C function inspired by Mario Pineda-Krch and his Package ‘GillespieSSA’. The code is available on request ([stockho@genethon.fr](mailto:stockho@genethon.fr) or [paldi@genethon.fr](mailto:paldi@genethon.fr)).

**Supplementary figure legends**

**Fig.S1 Correlation between the fluorescence level of reporter genes and the abundances of their mRNA-s.** The cytometry profiles of the high- and low-CFP and YFP fluorescent subclones are shown on the left panel. The normalized mRNA levels determined in the same cells using quantitative RT-PCR are shown on the right panel.

**Fig.S2 Bisulfite methylation analysis of the CMV promoter of the transgenes.** The methylation profile in cell subpopulations expressing both transgenes (right panel) and negative for both (left panel) were investigated. Each row of circles indicates a sequenced clone. Open circles indicate unmethylated CpG-s and black circles methylated sites.

**Fig.S3 Localization of the genomic integration sites of the transgenes.**

**Fig.S4 Quantitative RT-PCR analysis of the expression of genes at- and flanking the integration sites of the CFP- and YFP coding transgenes.** The levels of expression of these genes are similar in low- (grey bars) and high (black bars) reporter gene-expressing cell fractions as indicated on a normalized scale.

**Fig.S5: Computer simulation of the effects of protein stability on the evolution of the total fluorescence.** The simulated period of time was longer than 60 days. The results obtained with long half-lived mRNA and proteins are shown on the left side (panels A, C, E and G) and those with short half-lived proteins and mRNA on the right side (panels B, D, F and H). A and B show the state of the chromatin (open or closed). C through F panels represent changes in number of molecules (RNA and Protein) in a single cell and its daughter cells during 65 divisions (times in days). G and H represent the number of protein normalized by a hypothetical volume increasing linearly from 1 to 2 (that is to say the mean fluorescence level). Note that the global variance (NV = 1.26 for panel G and NV = 1.39 for panel H) is driven by the chromatin state.

**Fig.S6 Effect of transcription (left) and translation (right) inhibition on the recovery of fluorescence after whole cell photobleaching.** The number of cells examined in each experiment is indicated by “n”. Note the lack of recovery in both cases except a small and rapid initial increase due presumably to the termination of ongoing reactions or partial fluorescence recovery of some bleached molecules.

**S1 Movie 1. Time-lapse movie of the cell clone expressing the stable YFP and CFP proteins.** The YFP and CFP fluorescence was colored artificially in red and green for better visibility. The cells expressing both YFP and CFP are colored by the proportional mixture of the two colors. Note that the mother cells and their siblings have essentially the same color indicating similar levels of the two fluorescent proteins. One image was recorded every 10 minutes for five days.

**S2 Movie. Time-lapse movie of the cell clone expressing the unstable YFP and CFP proteins.** The YFP and CFP fluorescence was colored artificially in red and green for better visibility. The cells expressing both YFP and CFP are colored by the proportional mixture of the two colors. Note that the cells change fluorescence intensity during a single cell cycle. As a result, individual cell lineages cannot be tracked on the basis of the fluorescent protein expression level. One image was recorded every 20 minutes foe 3 days.
